# Comparison between High- and Low-Cost Transmission of Tele-Anesthesia in Japan

**DOI:** 10.1155/2018/9615264

**Published:** 2018-06-11

**Authors:** Yoh Sugawara, Tetsuya Miyashita, Yusuke Mizuno, Yusuke Nagamine, Tomoyuki Miyazaki, Ayako Kobayashi, Kentaro Tojo, Yasuhiro Iketani, Shunsuke Takaki, Takahisa Goto

**Affiliations:** Department of Anesthesiology, Yokohama City University Hospital, Yokohama, Japan

## Abstract

**Background:**

We previously reported a tele-anesthesia system that connected Sado General Hospital (SGH) to Yokohama City University Hospital (YCUH) using a dedicated virtual private network (VPN) that guaranteed the quality of service. The study indicated certain unresolved problems, such as the high cost of constantly using a dedicated VPN for tele-anesthesia. In this study, we assessed whether use of a best-effort system affects the safety and cost of tele-anesthesia in a clinical setting.

**Methods:**

One hundred patients were enrolled in this study. We provided tele-anesthesia for 65 patients using a guaranteed transmission system (20 Mbit/s; guaranteed, 372,000 JPY per month: 1 JPY = US$0.01) and for 35 patients using a best-effort system (100 Mbit/s; not guaranteed, 25,000 JPY per month). We measured transmission speed and number of commands completed from YCUH to SGH during tele-anesthesia with both transmission systems.

**Results:**

In the guaranteed system, anesthesia duration was 5780 min (88.9 min/case) and surgical duration was 3513 min (54.0 min/case). In the best-effort system, anesthesia duration was 3725 min (106.4 min/case) and surgical duration was 2105 min (60.1 min/case). The average transmission speed in the best-effort system was 17.3 ± 3.8 Mbit/s. The system provided an acceptable delay time and frame rate in clinical use. All commands were completed, and no adverse events occurred with both systems.

**Discussion:**

In the field of tele-anesthesia, using a best-effort internet VPN system provided equivalent safety and efficacy at a better price as compared to using a guaranteed internet VPN system.

## 1. Introduction

Telemedicine is defined as the use of medical information exchanged from one site to another via electronic communications to improve patients' clinical health status [1]. Improvements in telecommunication technology have increased transmission speed, allowing stable transfer of bulk data and improved quality of telemedicine and access to health care [2]. Telemedicine is of two basic types, synchronous and asynchronous. The synchronous system supports immediate interaction and responses within an acceptable wait period (not more than a few minutes). The major advantage of a synchronous system is that it provides a clinical decision or advice while seeking additional information or data. The areas of telemedicine that need synchronous systems are intensive care, emergency medicine, and mental health [3]. Asynchronous systems are characterized by time separation between one phase of the process and another [3]. Asynchronous telemedicine is used in medical fields that do not require immediate interaction and responses, such as telepathology, teleradiology, teledermatology, and wound care [3]. Earlier, limited telecommunication infrastructure and the high cost of peripheral devices were constraints to the development of synchronous systems. However, because of the recent large increase and development in bandwidth availability, telemedicine is now seeing a greater shift toward synchronous systems [3, 4].

In Japan, the shortage and uneven distribution of medical resources, especially in unpopulated areas, is a major issue [5]. The shortage of anesthesiologists is particularly a serious problem, resulting in compromised safety of patients undergoing surgery. One solution to this problem is the “tele-anesthesia system,” that is, an online anesthesia system that assists anesthesiologists and nurses. This system might reduce the workload of the anesthesiologist. The tele-anesthesia system requires very high synchronization due to the combination of time critical interactions and constantly changing information or data, and hence, it is associated with several technical challenges. We previously reported a tele-anesthesia system that connected Sado General Hospital (SGH) located on Sado Island to Yokohama City University Hospital (YCUH, Yokohama, Japan) using a dedicated virtual private network (VPN) that guarantees the quality of service (guaranteed system) [6]. The previous study showed that a higher bandwidth shortened delay time and increased the frame rate and that there were no adverse events related to the tele-anesthesia system during the study period. At the same time, the study revealed certain issues that needed to be resolved, such as the high cost of a dedicated VPN required for constant use of a tele-anesthesia system. The prohibitive cost can be reduced by using a best-effort delivery network system (best-effort system) that is cheaper than a guaranteed system but does not ensure the quality of service.

In this study, we evaluated the differences between the two transmission methods, the best-effort system and guarantee system, in terms of patient safety and cost of transmission, as well as the accuracy of command transmission during tele-anesthesia in a clinical setting.

## 2. Methods

A VPN (Arcstar Universal One, NTT Communications, Japan) was established between SGH and YCUH, as previously described [6]. The transmission speed was fixed using the guaranteed VPN at 20 Mbit/s (October 2013 to April 2015) in 65 operations, and a best-effort internet VPN (FLET's Hikari, NTT communications, Japan) was used (April 2015 to October 2015) in 35 operations (Table 1). This study was carried out without randomization for patients who meet inclusion criteria. Target surgery was general surgery, urology, orthopedics, and gynecology.

### 2.1. Monitors and Transmission between SGH and YCUH

**Figure 1:**
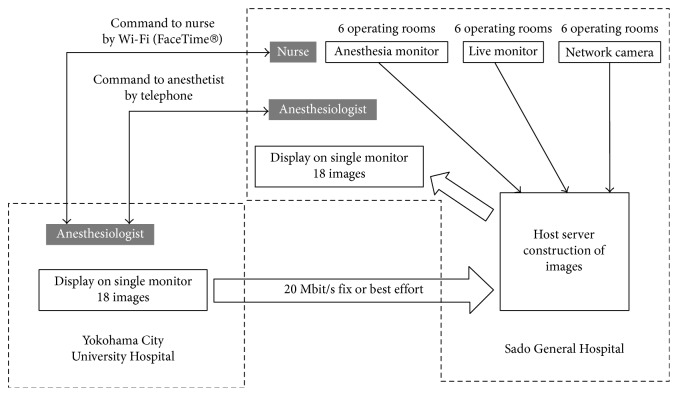
Transmission and communication systems between Sado General Hospital (SGH) and Yokohama City University Hospital (YCUH). In this study, we used three communication tools. We assessed the patients' vital signs and the progress of surgery using images transmitted via VPN, and communications between SGH and YCUH involved the use of FaceTime using a Wi-Fi connection and a telephone.

**Figure 2:**
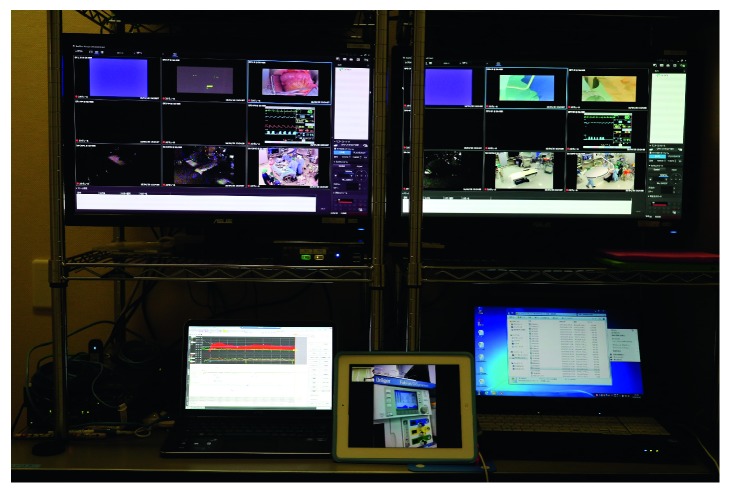
Monitors and tablet computer used at Yokohama City University Hospital (YCUH). There were two monitors for the images received from Sado General Hospital (SGH). The anesthesiologist at YCUH could synchronously observe 18 images from SGH.

SGH has six operating theatres, and 3 surgical operations under general anesthesia could be performed at the same time. Data from the anesthesia monitor (Dynascope DS-8500, Fukuda Denshi, Japan, 640 × 480 pixels, 5 frames per second), surgical site monitor (AG-MDC10G, Panasonic, Japan, 640 × 480 pixels, 5 frames per second), and operating room monitors (SNC-EP520, SONY, Japan, 640 × 480 pixels, 5 frames per second) in all six rooms were displayed on a single monitor (18 images) via a host server (Figure 1). We used a personal computer (ELITE 8300 US/CT, HP Compaq, USA), on which we installed network camera recording software (RealShot Manager, Sony, Japan) to reconstruct and display all the images on a single monitor at SGH. At YCUH, two display monitors (each display showed nine images) and two PCs (ELITE 8300 US/CT, HP Compaq, USA), on which RealShot Manager was installed, were used to reconstruct and display the images in a control room. The images that were imported to the host server at SGH were transmitted to YCUH via the VPN (guaranteed system or best-effort system). Anesthesiologists at YCUH could synchronously observe the 18 images from SGH using these systems [6] (Figure 2).

### 2.2. Communication Systems

Wi-Fi connections were set up in the operating theatres prior to the study. Free videoconferencing software (FaceTime, Apple, USA) was used for communications between anesthesiologists at YCUH and nurses at SGH. For FaceTime communications, we used an iPod touch device (4th generation, Apple, USA) in the operating theatre at SGH and an iPad (4th generation, Apple, USA) at YCUH. All devices had the most recent operation system installed. In the event that FaceTime disconnected, a telephone was used instead. For anesthesiologist to anesthesiologist communications between YCUH and SGH, a hands-free telephone was used (Figure 1) [6]. During anesthesia induction and extubation, the anesthesiologist at YCUH would need to use both the iPad and the telephone simultaneously to communicate with both the nurse and the anesthesiologist at SGH at the same time.

### 2.3. Roles of Nurses and Anesthesiologists at SGH

All drug injections and procedures were performed according to commands from the anesthesiologist at YCUH. The anesthesiologist at SGH performed mask ventilation, endotracheal intubation and extubation, and all other procedures that require a medical license. A nurse performed other procedures, including anesthetic and vasoactive drug injections [6].

### 2.4. Roles of the Anesthesiologist at YCUH

The anesthesiologist at YCUH provided commands to the anesthesiologist and nurse at SGH. These included all commands for the induction of general anesthesia, such as drug injection, mask ventilation, tracheal intubation, and change in body position for surgery. After confirming the safe induction of general anesthesia and patient stability, the anesthesiologist at SGH left the operating theatre and waited for the command from the anesthesiologist at YCUH in the control room. During the maintenance of general anesthesia, the anesthesiologist at YCUH gave instructions to the nurse at SGH regarding the care of the patient. If an adverse event, such as hypotension/hypertension (systolic blood pressure <80 mmHg or >160 mmHg), bradycardia/tachycardia (heart rate <50 bpm or >120 bpm), or low arterial saturation (SpO_2_ < 95%), continued for 5 min despite the nurse administered adequate drugs, the anesthesiologist at YCUH called the anesthesiologist at SGH to treat the event. After the surgical procedure was completed, the anesthesiologist at YCUH called the anesthesiologist at SGH to reenter the operating theatre and provided instructions on all procedures, including emergence from general anesthesia, suction, extubation, and any other required procedure until the patient had been shifted from the operation theatre [6].

### 2.5. Command Completion and Measurement of Transmission Speed

All commands from the anesthesiologist at YCUH were recorded in the anesthesia records at SGH, and the number of commands was counted by the anesthesiologist at YCUH. For best-effort VPN, transmission speed was measured and recorded at YCUH.

### 2.6. Clinical Practice

This study was approved by the appropriate ethics committees of Sado General Hospital and Yokohama City University Hospital. Written informed consent was obtained from 100 adult patients at SGH who satisfied the inclusion criteria (18 years old or above, ASA physical status 1-2, scheduled to undergo surgery, expected blood loss of less than 500 ml, anticipated duration of surgery less than 3 hours). There were no dropouts during the course of this study. The transmission speed was fixed at 20 Mbit/s with the guaranteed system (October 2013 to April 2015) in 65 operations, while a best-effort internet VPN was used (April 2015 to October 2015) in 35 operations.

## 3. Results

One hundred patients were enrolled in the study. The patients' background and clinical data are shown in Table 1. The total duration of anesthesia was 9505 min (mean, 95.0 min/case), and the surgical duration was 5618 min (mean, 56.2 min/case). In the guaranteed system, duration of anesthesia was 5780 min (mean, 88.9 min/case) and surgical duration was 3513 min (mean, 54.0 min/case). In the best-effort system, duration of anesthesia was 3725 min (mean, 106.4 min/case) and surgical duration was 2105 min (mean, 60.1 min/case). The numbers of commands given for different types of drugs and procedures are shown in Table 2 (for nurses) and Table 3 (for anesthesiologists). A total of 1976 commands were given to nurses via FaceTime, and 965 commands were given to anesthesiologists via the telephone. All 2941 commands were completed. Although there were 53 min of FaceTime disconnections (Table 1), the telephone for nurses was never used. In one case, in which a patient undergoing transurethral ureterolithotomy had drug-resistant hypotension and bradycardia that continued for 5 min, the anesthesiologist at YCUH called the anesthesiologist at SGH to reenter the operating theatre.

**Figure 3:**
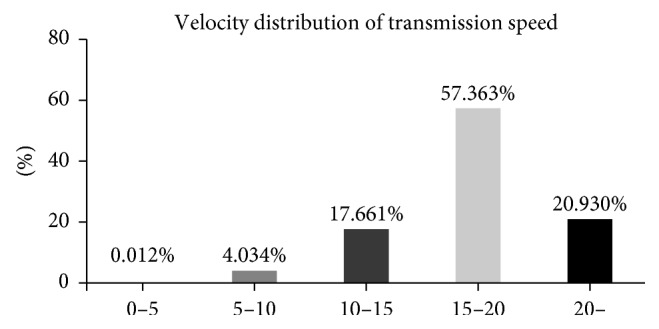
Distribution of transmission speed using the best-effort system. Average transmission speed was 17.3 ± 3.8 Mbit/s, and the modal class was 15 to 20 Mbit/s (57.363%). Transmission speed less than 5 Mbit/s was only infrequently seen.

In this study, we used two types of transmission speeds, the guaranteed system in which transmission speed was fixed at 20 Mbit/s and the best-effort system with a variable transmission speed. The distribution of speed of transmission in the best-effort system is shown in Figure 3. Average transmission speed was 17.3 ± 3.8 Mbit/s, and the modal class was 15 to 20 Mbit/s (57.363%). There were few instances of a transmission speed less than 5 Mbit/s in this study (0.012%). No artefacts or command errors were observed at any transmission speed.

## 4. Discussion

In this study, we demonstrated that, in the field of tele-anesthesia, use of a best-effort internet VPN system provides equivalent safety and efficacy, and at a better price, as compared to using a guaranteed internet VPN system. We performed 100 cases of tele-anesthesia, including a tele-anesthesia duration of 5780 min (88.9 min/case) using the guaranteed VPN system and 3725 min (106.4 min/case) using the best-effort system. Although there are a few case reports of tele-anesthesia, the data size and methods of tele-anesthesia in the previous studies were limited [6–8]. Our study is the largest study on tele-anesthesia in terms of sample size to be reported in the past decade.

The aim of this study was to assess whether a difference in the transmission method used, the best-effort system or guaranteed system, affects patient safety and the cost of tele-anesthesia in a clinical setting. We previously reported a pilot study about teleconnection of SGH to YCUH to test the efficiency of the tele-anesthesia system [6]. Our previous study showed that the clinically available transmission speed with a guaranteed VPN system was never lower than 5 Mbit/s. If transmission speed is lower than 5 Mbit/s, the delay time in communication is prolonged to a clinically unacceptable level [6]. In this study, we measured transmission speed using a best-effort VPN system and found that the duration of transmission speed was virtually never lower than 5 MBit/s (0.012%) (Figure 3). These results indicate that, in terms of transmission speed, use of a best-effort system in clinical practice provides an acceptable delay time and frame rate. Consequently, no artefacts were observed at any transmission speed in the present study.

Although dedicated VPNs that guarantee the quality of service are robust and provide a safe transmission system for tele-anesthesia, the cost of a dedicated VPN with a speed of 20 Mbit/s is too expensive for continuous use during tele-anesthesia (372,000 JPY per month: 1 JPY = approximate US$0.01). In contrast, a best-effort system that uses a public line costs 25,000 JPY per month, which is much lower than the cost of a guaranteed system. However, contractual coverage limits the quality of service, including the transmission speed, with the best-effort system.

Telemedicine is a growing field that includes a variety of applications and services using two-way video, email, smart phones, wireless tools, and other forms of telecommunication technologies [1]. In this study, we used three communication tools. We assess the patients' vital signs and progress of surgery by images transmitted via VPN, and the communication tools between SGH and YCUH were FaceTime (which required a Wi-Fi connection) and the telephone. No adverse events related to tele-anesthesia were observed in this study. No artefacts were observed in the images transmitted via VPN. The nurses received a total of 1976 commands via FaceTime, and 965 commands were issued to anesthesiologists via the telephone. All of the 2941 commands were completed (Table 2). In one case, in which a patient had drug-resistant hypotension and bradycardia that continued for 5 min, the anesthesiologist at YCUH called the anesthesiologist at SGH to reenter the operating theatre. The patient recovered with fluid resuscitation (650 ml of crystalloids), intravenous injection of atropine (0.5 mg), and phenylephrine (0.4 mg). These results show that a combination of these three technological tools facilitates the effective performance of tele-anesthesia.

Our study has some limitations. The sample size in this study was too small to assess patient safety when using this system. We excluded cases deemed difficult for anesthesia, such as those with ASA status > 3, due to the expectation of unstable vital signs, prolonged surgical time > 3 hours, or expected massive bleeding.

## 5. Conclusion

The tele-anesthesia system might be part of the solution to the problem of medical resource shortages. The recent development of small, low-cost devices might enhance the safety and feasibility of tele-anesthesia. Recently, automated anesthesia using closed-loop technology has been reported [9]. A combination of safer tele-anesthesia and automated anesthesia is expected in the future.

## Figures and Tables

**Table 1:** 

	Total (*N*=100)	Fix VPN (*N*=65)	Best-effort VPN (*N*=35)
*Characteristics*			
Male	63	45	18
Female	37	20	17
Age	66.8	70.2	60.5
Body weight	59.1	58.4	60.5
ASA	I: 48 II: 52	I: 26 II: 39	I: 22 II: 13
Anesthesia time	9505	5780	3725
Operation time	5618	3513	2105

*Command*			
Duration of commands to 2 operation rooms simultaneously (min)	428	428	0
Duration of FaceTime disconnection (min）	53	40	13
Anesthetist call	1	1	0

*Type of surgery*			
General surgery	23	13	10
Urology	36	29	7
Orthopedics	37	19	18
Gynecology	4	4	0

**Table 2:** 

	Total		Fix VPN		Best-effort VPN	
	Command	Command/case	Command	Command/case	Command	Command/case
*Anesthetic drugs*						
Propofol	100	1.18	75	1.15	42	1.20
Fentanyl	133	1.56	110	1.69	48	1.37
Remifentanil (civ)	297	3.49	235	3.62	106	3.03
RB (IV)	135	1.59	97	1.49	65	1.86
NSAIDs	47	0.55	28	0.43	32	0.91
Sugammadex	87	1.02	67	1.03	35	1.00

*Vasoactive drugs*						
Ephedrine	66	0.78	50	0.77	28	0.80
Phenylephrine (IV)	25	0.29	12	0.18	15	0.43
Phenylephrine (civ)	46	0.54	37	0.57	10	0.29
Atropine	43	0.51	31	0.48	20	0.57
Others	3	0.04	3	0.05	0	0.00

*Other drugs*						
Antibiotics	84	1.0	64	1.0	35	1.00
Local anesthesia	32	0.4	18	0.3	18	0.51

*Other procedures*						
div speed	167	2.0	118	1.8	77	2.20
Volatile Anesthetics	219	2.6	171	2.6	78	2.23
Ventilator	199	2.3	155	2.4	63	1.80
Alarm setting	32	0.4	29	0.4	4	0.11

*Total*	1715		1300		676	

civ: continuous venous infusion.

**Table 3:** 

	Total		Fix VPN		Best-effort VPN	
	Command	Command/case	Command	Command/case	Command	Command/case
*Procedures*						
Mask ventilation	85	1.0	65	1.0	35	1.00
Intubation	87	1.0	65	1.0	38	1.09
Extubation	85	1.0	65	1.0	35	1.00
Suction	110	1.3	66	1.0	82	2.34
Postoperative oxygen	85	1.0	65	1.0	35	1.00

*Others*						
BIS	83	1.0	63	1.0	35	1.00
Confirmation of awareness	85	1.0	65	1.0	35	1.00
Confirmation of airway	85	1.0	65	1.0	35	1.00
Position change	96	1.1	79	1.2	37	1.06

*Total*	801		598		367	

## Data Availability

All data, except for Figure 3, were described in all of the tables of the article. Data of Figure 3 are available from the corresponding author upon request.

## Abbreviations

SGH: Sado General Hospital
YCUH: Yokohama City University Hospital
VPN: Virtual private network
JPY: Japanese yen
ASA: American Society of the Anesthesiologists.

## References

[1] Association AT (2013). *What is Telemedicine*.

[2] Alvi S. A., Afzal B., Shah G. A., Atzori L., Mahmood W. (2015). Internet of multimedia things: vision and challenges. *Ad Hoc Networks*.

[3] Wilson L. S., Maeder A. J. (2015). Recent directions in telemedicine: review of trends in research and practice. *Healthcare Informatics Research*.

[4] Ward M. M., Jaana M., Natafgi N. (2015). Systematic review of telemedicine applications in emergency rooms. *International Journal of Medical Informatics*.

[5] Akiyama M., Yoo B.-K. (2016). A systematic review of the economic evaluation of telemedicine in Japan. *Journal of Preventive Medicine and Public Health*.

[6] Miyashita T., Mizuno Y., Sugawara Y. (2015). A pilot study of tele-anaesthesia by virtual private network between an island hospital and a mainland hospital in Japan. *Journal of Telemedicine and Telecare*.

[7] Wehbe M., Arbeid E., Cyr S. (2014). A technical description of a novel pharmacological anesthesia robot. *Journal of Clinical Monitoring and Computing*.

[8] Fiadjoe J., Gurnaney H., Muralidhar K. (2009). Telemedicine consultation and monitoring for pediatric liver transplant. *Anesthesia and Analgesia*.

[9] Simpao A. F., Galvez J. A. (2015). Current and emerging technology in anesthesia. *Anesthesiology*.

